# Gestures can help children learn mathematics: how researchers can work with teachers to make gesture studies applicable to classrooms

**DOI:** 10.1098/rstb.2023.0156

**Published:** 2024-10-07

**Authors:** Amanda Seccia, Susan Goldin-Meadow

**Affiliations:** ^1^ Division of Social Sciences, Psychology, University of Chicago, Chicago, IL 60637, USA

**Keywords:** gesture, teaching, learning, retention, generalization

## Abstract

The gestures we produce serve a variety of functions—they affect our communication, guide our attention and help us think and change the way we think. Gestures can consequently also help us learn, generalize what we learn and retain that knowledge over time. The effects of gesture-based instruction in mathematics have been well studied. However, few of these studies are directly applicable to classroom environments. Here, we review literature that highlights the benefits of producing and observing gestures when teaching and learning mathematics, and we provide suggestions for designing research studies with an eye towards how gestures can feasibly be applied to classroom learning.

This article is part of the theme issue ‘Minds in movement: embodied cognition in the age of artificial intelligence’.

## 1. What is gesture and what are its functions?

Across all cultures, people use hand movements that represent ideas when they talk—in other words, they gesture [1–4]. Why does everyone, young and old, gesture? One way to answer this question is to ask whether gesture has a function? It turns out that it has many.

Gesture attracts and directs our attention. When children are taught how to solve a mathematics problem with gesture-based instruction, they not only follow the words that accompany the teacher’s gestures, but they also glean more information from those words than children who follow their teachers’ words but without gestures [5]. Observing gestures can help learners look in the right place and get meaning from that place.

Gesture helps us recall information. After watching a series of videos, adults who gesture while explaining what they see remember more than adults who do not gesture [6]. Gestures may help us remember more because they lighten our cognitive load. For example, adults [7] and children [8] who are asked to remember letters while also explaining how they solved a mathematics problem remember more letters when they gesture during their explanations than adults and children who do not gesture. Producing gestures can reduce the cognitive effort required to keep information in memory.

Gestures can change the way we think. When they were told to gesture during their explanations, children who were asked to explain whether stealing is worse than cheating placed their gesturing hands in locations in space that represent different perspectives. These children displayed more advanced moral reasoning in their spoken explanations than children told not to gesture by taking perspectives other than their own into account [9]. By externalizing our thoughts, our gestures can ground ideas in space, which has the potential to expand our perspectives.

Gesture brings a second modality into communication, which can impact how we think and learn [10], and it brings the body into learning [11]. Gesture impacts cognition in multiple ways, all at the same time. In this sense, gesture is a ‘perfect storm’ [2]; it is a powerful, natural, confluence of factors that alter an event—in this case, a learning event—and, as a result, has a profound effect on our thoughts and interactions. It is therefore not surprising that gesture can play a role in teaching and learning all subjects, particularly mathematics.

## 2. What role can gesture play in learning mathematics?

Gesture has the potential to play a role in learning in several ways. First, the gestures children produce when solving and explaining a mathematics problem can reveal their understanding of that problem. If teachers can ‘read’ these gestures, they will have access to thoughts that may not appear in a child’s speech. If they then teach to those thoughts, the child will get instruction tailored to their current knowledge of the problem. Second, the gestures children produce while solving a mathematics problem can promote learning, presumably because they engage in the cognitive processes described in the previous section. Finally, the gestures that teachers produce when explaining mathematical concepts can influence what children take from a lesson and thus promote learning. We broadly define ‘mathematics’ as any skills related to numeracy, including conservation of numbers, spatial reasoning, measurement, balance and algebraic thinking.

### (a) Children’s gestures can predict learning

The gestures children produce when explaining how they solved a mathethamatics problem often convey the same information as the speech accompanying those gestures; their gestures *match* their speech. But, at times, children’s gestures add to information about the problem conveyed in speech; their gestures *mismatch* their speech [1,12]. Children who produce gesture–speech mismatches on a mathematics problem are ready to learn that problem, and their gestures let their teachers know.

#### (i) Children’s gestures reveal when they are ready to learn

Let us take an example from a conservation of number task [13]. Two rows have an equal number of checkers. One row is spread out, and the child’s task is to say whether the rows still have the same number. One child says that the top row has more checkers because ‘you moved them’. However, at the same time, the child points to the first checker in the top row and then to the first checker in the bottom row; he then points to the second checker in the top row, the second checker in the bottom row and so on. The child is using his hands to indicate one-to-one correspondence between the checkers in the two rows, an important step in understanding the conservation of numbers [12]. The child’s speech suggests that he has no understanding of conservation. His hands suggest otherwise.

Children who produce gesture–speech mismatches on number conservation problems are likely to improve their understanding of conservation when given instruction—more likely than children who produce only gesture–speech matches (in this case, the matching child gestures the movement used to spread the row out) [12]. This example includes one incorrect strategy (in speech) and one correct strategy (in gesture). However, even children who produce two incorrect strategies on a problem—one in gesture and the other in speech—are also ready to learn the problem. Gesture–speech mismatches convey information about a child’s state of knowledge about a particular problem.

Mismatches predict learning in many areas that are taught in classrooms. Understanding mathematical equivalence (i.e. understanding that two sides of an equation must be equal, often tested with problems of the following type, 5 + 4 + 3 = __ + 3) is a crucial part of algebra, which predicts success in other STEM fields [14–17]. Some children produce gesture–speech mismatches when solving these problems. For example, when asked to explain their incorrect answer, 16, one child said, ‘I added up all the numbers’. At the same time, the child pointed to the two numbers on the left side of the equation that can be grouped together and added to find the number that belongs in the blank (e.g. 5 and 4 in the example). As in the conservation problems, children who produce mismatches are more likely to improve their understanding of mathematical equivalence after instruction than children who produce only matches (e.g. they point at all four numbers while saying they added all of the numbers [18,19]. Although children who mismatch do not display a correct understanding of the problem in their speech, their hands indicate that they know more than they say.

Mismatches also predicted learning in a study where children were asked to explain how they solved a balancing task [20]. Understanding balance can help develop spatial awareness and the relationship between objects in space. Children were asked to balance wooden beams on a fulcrum. Some of the beams were symmetric, or equally weighted on either end (e.g. some beams had one block on both ends or two blocks on both ends). Other beams were asymmetric or did not weigh the same on each end (e.g. some beams had one block on one side or two blocks on one side). About one-third of the children produced mismatches when they explained how they made the beams balance. For example, a child might verbally explain that a beam balances ‘because it is in the middle’, but they may accompany their speech with a weight gesture (i.e. the child holds their fingers together, pointing downwards, and moves their hand up and down at one end of the beam). Children whose gesture and speech were mismatched during the pre-test were more likely to improve on the post-test (i.e. they successfully balanced asymmetrical beams by shifting the centre point of the beam of the fulcrum) than those whose speech and gesture matched. This study provides further evidence that mismatches can predict learning.

Gesture is a reliable indicator of a student’s knowledge state. It can therefore be used by a teacher to figure out what a child’s implicit understanding of a problem is and to fashion instruction with that level of understanding in mind. But, of course, this process will work only if teachers can read children’s gestures.

#### (ii) Teachers can read children’s gestures

Understanding the information children convey with their gestures may be especially beneficial for teachers. Teachers are trained to observe and analyse student responses and explanations, but do they glean information from their students’ gestures? To test this hypothesis, Alibali *et al*. [21] asked 20 teachers to watch videos of individual children explaining their answers to mathematics equivalence problems and then describe the child’s reasoning. When children produced mismatches, teachers often described the information conveyed uniquely in the child’s gestures. Teachers do have access to the thoughts children hide in their hands.

Do teachers glean information from children’s gestures because they are trained to observe learning processes, or can most adults pick up information from a child’s gestures? Alibali *et al.* [21] showed the same videos to 20 undergraduate students and found that the undergraduates were also able to describe the information that children conveyed uniquely in gesture. In a study of conservation, Goldin-Meadow & Sandhofer [22] video-recorded children as they explained their answers to the conservation task described earlier. They asked adults to watch the videos and to indicate which explanations each child gave. In addition to listing the explanations children gave in speech, many adults mentioned explanations conveyed *only* through gestures. For example, while watching a child who argued for non-conservation in speech but displayed an understanding of conservation in gesture, one adult said that the child understood that the number of checkers in one row matched the number of checkers in the other row ‘even though he wasn’t verbalizing it’. Both teachers and untrained adults can gather information from children’s spontaneous, unedited, fleeting gestures even when that information is not conveyed in speech. However, gleaning information from gestures may come at a cost—observers may miss some of the information conveyed verbally when they process gestures ([22] although this decrease can be improved with training; see Kelly *et al.* [23]).

### (b) Producing gestures can promote learning

Children can learn from producing their own gestures during a lesson. Cook & Goldin-Meadow [24] showed third- and fourth-grade children how to solve a series of mathethamatics equivalence problems. Some children were taught with speech alone: when teaching how to solve ‘4 + 6 + 3 = __ + 3’, the experimenter said, ‘I want to make one side equal to the other side. See, 4 plus 6 plus 3 equals 13, and 10 plus 3 equals 13. That’s why I put 10 in the blank’. Other children were taught with speech and gesture. The experimenter demonstrated the equalizer strategy and swept their left hand under the left side of the equation when they said ‘one side’ and swept their right hand under the other side of the equation when they said ‘the other side’. When the children practised problems on their own and explained their answers, they produced the speech or speech and gestures they had learned. The children who observed gestures during instruction performed better on the post-test than those who only heard the speech, but those who also produced their own gestures performed significantly better than those who did not [24]. Producing their own gestures is an effective way for children to learn.

This work provides an important first step in showing that gesture plays a role in learning. However, children not only need to learn the problems on which they are taught, but they also need to transfer that knowledge to new contexts (generalization) and remember what they have learned over time (retention).

#### (i) Producing gesture promotes generalization

An important aspect of learning is transferring the knowledge gained to a new context. Gesturing can help here. Novack *et al*. [25] gave children a lesson with mathematical equivalence problems written on a whiteboard (e.g. 5 + 4 + 6 = __ + 6) and with magnetic number tiles placed over each number. Children were taught the equalizer strategy in speech and one of three grouping strategies using their hands: (i) Pick up the two number tiles that need to be added together to solve the problem (i.e. the 5 and the 4), and then hold the tiles over the blank (action strategy). (ii) Gesture picking up the same magnetic number tiles without touching them and gesture holding that hand over the blank (concrete gesture). (iii) Produce a V-shaped point at the 5 and 4 and then point at the blank (abstract gesture).

Children in all conditions learned how to solve the problem, but only children in the two gesture conditions generalized that knowledge to new formats (referred to as transfer problems)—one where the second two numbers must be added to solve the problem, rather than the first two (e.g. 5 + 4 + 6 = 5 + __), and another where there are no duplicated numbers so no grouping strategy will work (e.g. 5 + 4 + 6 = __+ 3). Both action and gesture can help children learn how to solve a problem, but only gesture helps them generalize that knowledge to a new problem format. Action may impede generalizing by getting children to fixate on the manipulated objects (in this case, the magnetic tiles), preventing them from transferring the knowledge they gained during the lesson [25]. At the same time, gestures may promote abstraction.

#### (ii) Producing gesture promotes retention

Teaching children a gesture to produce during a lesson not only helps them learn the lesson, but it also helps them retain what they learned. In a study by Cook *et al*. [26], children produced the equalizer strategy for solving mathematical equivalence problems in speech and/or gesture. One group produced the strategy in both gesture and speech, one in gesture alone and one in speech alone. The groups that gestured during the lesson retained the knowledge they had gained for at least four weeks. The group that learned the spoken strategy without gestures learned how to solve the problem but did not retain what they learned. Even when children are taught to produce one strategy in gesture and a *different* strategy in speech during a mathethamatics lesson (i.e. a mismatch), they still retain the knowledge they gained four weeks later [27]. Producing gestures during the learning process benefits retention.

### (c) Observing gestures can promote learning

Children learn not only from producing their own gestures but also from watching others produce gestures. To examine this process in a controlled setting, Singer & Goldin-Meadow [28] taught children how to solve mathethamatics equivalence problems by varying whether their instruction contained a gesture and, if so, whether the gesture conveyed the same strategy as the speech the teacher used or a different strategy. Some groups heard only the equalizer strategy in speech (speech only); some heard the equalizer strategy in speech and saw the equalizer strategy in gesture (speech and matching gesture) and some heard the equalizer strategy and saw the add–subtract strategy (add the numbers on the left side of the equation and subtract the number on the right) in gesture (speech and mismatching gesture). Children learned better when they saw gestures than when they heard only speech. But the best instruction was speech and *mismatching* gesture. To control for the number of strategies the child was taught, an additional group of children heard the equalizer strategy and the add–subtract strategy in speech with no gesture. These children showed very little progress at all. In other words, getting two strategies in instruction was effective only when one strategy was in speech and the other in gesture. Having two ways to solve a problem improves children’s chance of grasping the underlying concept [10,29], but only when one of those ways is expressed in gesture (at least in the mathematical equivalence context).

#### (i) Observing gesture promotes child retention

By incorporating gestures into lessons, teachers can help children perform well right after the lesson and also retain what they learned over time. Congdon *et al*. [29] tested children right after a mathethamatics lesson and also 24 h and four weeks later. All of the children received two strategies in their lessons (equalizer and add–subtract), but some heard both strategies in speech (speech alone); some heard equalizer in speech followed by add–subtract in gesture (speech *then* gesture); some heard equalizer in speech presented at the same time as add–subtract in gesture (speech *and* gesture). Children in all three conditions performed equally well right after the lesson. But only children taught with gestures simultaneously presented with speech retained their knowledge 24 h and four weeks later. In fact, the two other groups lost ground on the delayed tests. Seeing a teacher’s gesture can make learning last, particularly when that gesture is produced simultaneously with speech.

#### (ii) Observing gesture promotes child generalization

In addition to testing retention of mathethamatics equivalence, Congdon *et al*. [29] also tested the children’s ability to generalize what they learned to problems in a new format. At each testing time, children were given problems comparable to those on which they were taught and problems that required them to extend the knowledge they had gained. Children who received simultaneous speech and gesture instruction performed significantly better than the other two groups on the transfer problems at both 24 h and four weeks after instruction.

Including gestures in a lesson not only leads to success on the taught problems but also helps children generalize what they learned to new problem formats, again when that gesture is produced simultaneously with speech. Using gestures during instruction promotes long-lasting, flexible learning, which is a central goal of education.

### (d) How can producing and observing gestures work together to promote learning?

The fact that children and teachers both gesture in the classroom provides an opportunity for a conversation that extends beyond speech. We know that teachers can glean information from children’s gestures, but do they use this information to guide their own instruction? Goldin-Meadow & Singer [30] asked eight teachers to observe children individually explaining how each solved mathethamatics equivalence problems. The teachers were then asked to teach each of the children they saw how to solve the problems. Their instruction varied as a function of the child’s gestures. The teachers used more different types of problem-solving strategies when teaching children who produced gesture–speech mismatches than when teaching children who only produced gesture–speech matches. Interestingly, they also produced more of their own gesture–speech mismatches when teaching children whose gestures mismatched than when teaching children whose gestures matched. The teachers’ mismatches were not copies of the children’s mismatches—the teachers’ mismatches always contained two correct, different strategies; the children’s mismatches always contained at least one, and sometimes two, incorrect strategies. This work shows that teachers, perhaps without knowing, adjust instruction and their own gestures based on their (implicit) observation of student gestures.

The children who received multiple strategies and gesture–speech mismatches from the teacher in the Goldin-Meadow & Singer [30] study were particularly likely to learn the task. But they may have learned because they were ready to learn, not because of the teacher’s instruction. To find out whether the teachers’ spontaneous choices were good for instruction, Singer & Goldin-Meadow [28] gave a group of children two different problem-solving strategies in speech (equalizer and add–subtract); another group was given two different problem-solving strategies, one in speech (equalizer) and the other in gesture (add–subtract), that is, a gesture–speech mismatch; and another group was given the same strategy in speech and in gesture (equalizer), that is, a gesture–speech match. The teachers in the Goldin-Meadow & Singer [30] study were right to give children mismatches in mathematical equivalence instruction—children who received mismatching instruction in the controlled study [28] improved the most. But the two strategies had to be in gesture and speech—giving children two different strategies entirely in speech did not help them improve. Children who received one strategy in speech and a matching strategy in gesture fell somewhere in between.

Gesture thus offers the opportunity for an undercurrent of give-and-take conversation in the classroom. Children gesture when explaining their ideas, and teachers pick up on those ideas. Teachers can then individualize their instruction to different learners, using their own gestures to do so [30]. In turn, when the teacher gestures, children increase their gesturing [26], completing the circle.

Observing and producing gestures both play a role in learning. However, producing gestures may be more beneficial to learners than observing gestures. The motor system is activated when the learner produces gestures, which promotes learning [31]. The motor system is also activated when the learner observes gestures [32], but the activation is stronger when learners produce a gesture than when they observe it [31]. Accordingly, in a meta-analysis of studies testing learning through either gesture production or observation, Dargue *et al.* [33] found larger effect sizes for gesture production than for gesture observation (see also Goldin‐Meadow *et al.* [34], who found the same pattern in a mental rotation task).

However, producing gestures and observing gestures are rarely compared within the same study. The exception is Congdon *et al*. [35] (see also Wakefield *et al*. [36]), who compared children producing their own gestures (or actions) with children observing a teacher’s gestures (or actions) and assessed learning on the taught mathematical equivalence problems, generalization and retention. Interestingly, they found no differences between observing gestures and producing gestures for children who produced their own gestures prior to instruction—children learned just as much from observing gesture as from producing it, and they generalized and retained what they had learned just as much from observing gesture as from producing it. The children also learned, generalized and retained equal amounts from observing action and producing action. However, replicating previous work [25,29], observing or producing *gesture* both led to better generalization and retention than observing or producing *action*.

Interestingly, the findings were different for children who produced *no* gestures on the task prior to instruction. They showed the same patterns for *observing* gestures as children who gestured prior to instruction—they generalized and retained more from observing gestures than observing actions. But they differed in *producing* gestures—they generalized and retained more from doing *action* than doing *gesture*. This study points to possible individual differences in the extent to which gesture can be a useful teaching tool. As with other examples, children with limited content knowledge may not profit from gestures as much as children who have already developed foundational skills related to the problem [29]. Memory capacity can also be a factor in how much children benefit from gesture. Individuals with lower working memory tend to gesture more frequently than those with higher working memory [37–39]. However, individuals with higher visuospatial working memory are better at learning from gestures than those with lower visuospatial working memory [40]. We need to learn much more about whether there are individual differences in gesturing and, if so, how they are reflected in authentic learning environments.

## 3. How can translational research leverage the use of gesture in the classroom?

The role that gesture can play in learning mathethamatics may be particularly useful for teachers to know about. Helping teachers effectively produce, and observe, gestures in the classroom during mathethamatics instruction could be a feasible, low-cost way to enhance learning outcomes. We next summarize empirically supported ways in which gesture can benefit teachers and students.

Teachers can learn a great deal about their students’ understanding of a problem from watching their gestures. When children produce a gesture on a problem that conveys different information from the speech it accompanies (i.e. a gesture–speech mismatch), they are telling the world with their hands that they are ready to benefit from instruction on that problem [1]. Gesture–speech mismatch reflects readiness to learn when children gesture naturally [12] and when they are prompted to gesture [41]. Teachers can use this cue to target instruction to children whose gestures show that they are ready to profit from it.

Teachers can also encourage students to gesture. We might have guessed that telling children to gesture would get them to focus on the relation between their gestures and speech, encouraging them to produce only gesture–speech matches. However, telling children who had just explained their answers to a mathematical equivalence problem to gesture on the next batch of explanations not only brought out gestures but also brought out mismatching gestures. Moreover, many of those mismatching gestures conveyed correct problem-solving strategies that the children had not yet expressed in speech. When these newly minted mismatching children were then given instruction in mathematical equivalence, they learned how to solve the problem more readily than children who were told not to gesture and therefore produced no mismatches [41].

Teachers can strategically produce gestures to promote learning. As noted earlier, children pay attention to instructors’ hands [5], which means that teachers can use their gestures to help children focus on essential components of a mathethamatics problem. By being more intentional with their gestures, teachers can encourage children to identify key parts of a mathematical concept. Teachers can also produce their own gesture–speech mismatches during instruction. Moreover, producing two different but correct strategies, one in gesture and the other in speech, helps children learn more efficiently [28].

Many studies have validated the benefit of using gestures for mathethamatics instruction. However, most of these studies have been conducted in one-on-one interactions, typically in an extra room or hall at the school. Do the benefits of producing and observing gestures during teaching and learning mathethamatics translate to classrooms?

### (a) What must researchers understand to conduct translational work?

Before the effects of gesture can be adequately tested in—and applied to—naturalistic (or authentic) learning environments, researchers must first develop effective ways of collaborating with educators. Historically, when researchers conduct classroom-based studies, they often collect data and leave with minimal interaction with teachers and students. In other words, there are few direct benefits for the teachers and students involved. It would be helpful if researchers were to take the time to understand (i) the systematic differences between research and teaching, (ii) the realities of classroom teaching, and (iii) how to develop flexible study designs conducive to classrooms.

#### (i) The systematic differences between teaching and research

Although teachers and researchers often share the common goal of trying to help children learn, the two groups work within separate systems. As a result, educators often report that empirical work related to teaching and learning is inaccessible, incomprehensible and not applicable to real classrooms [42]. The limited empirical work that ends up being disseminated to teachers often ends up being ineffective in the classroom, which leads to a regression to former practices [43]. To conceptualize and develop projects that translate to classrooms, we must first understand the underlying differences between teaching and research.

Teachers and researchers vary in the type of knowledge they value [44]. Teachers value the application of existing knowledge to guide their instructional practices. They rely on pedagogical knowledge that often derives from their daily experiences and observations. On the other hand, researchers aim to generate new knowledge and conduct studies designed to understand the mechanisms underlying learning, which are then published in scholarly, peer-reviewed journals. Teachers and researchers also differ in their reasons for seeking new knowledge. Teachers typically seek new knowledge to address specific teaching- and learning-related concerns that arise in their classrooms on a daily basis. Researchers seek new knowledge to test hypotheses and advance theories of how learning works [43]. The pace at which teachers and researchers operate also varies. Teachers often seek quick solutions that address real-time concerns [45]. Researchers can take years to address one question, which may, in the end, have inconclusive results [46,47].

The fundamental differences between teacher and researcher values and knowledge get in the way of productive collaboration. If researchers want to enhance learning outcomes, they must not only conduct long-term projects geared towards understanding the theoretical underpinnings of learning, but they must also focus on practical applications of their work that will result in sustained change that benefits student learning.

#### (ii) The realities of a classroom

Researchers often have the luxury of testing their teaching- and learning-related hypotheses on one participant at a time in a controlled, lab-based environment, free from distractions. However, authentic teaching and learning occur in classrooms that have many children, noises and unpredictable student behaviours. These uncontrollable factors occurring in classrooms are likely to impact learning. A teaching method might be effective in the lab, but when implemented in a classroom, other factors may prevent it from having an impact.

Most of the gesture studies described in this review have been conducted in schools. Specifically, these studies were conducted in one-on-one instructional situations when students were pulled out of their classrooms. This method is one step closer to conducting research in authentic learning environments in that the lessons are not taking place in a quiet lab, but it still does not take the nuances and realities of a classroom into account. Conducting studies in classrooms with all students (and not in hallways with individual students) is one way to ensure that research will translate into practice. However, classroom studies pose many difficulties. Teachers face a variety of challenges throughout the day—they must implement a curriculum, adhere to a district-wide pacing guide, prepare students for standardized tests and manage other cultural, racial and socio-emotional factors of teaching and learning. Teachers and administrators may consequently be hesitant to allow researchers to disrupt their daily routines to conduct research studies, particularly if those studies are not likely to lead to useful changes in the classroom. To work together with teachers, researchers need to develop flexible methods of investigation that better reflect realistic learning environments.

#### (iii) Developing flexible study designs

When conducting experimental studies for publication, researchers often use methods that are rigid and inflexible. The experimenter’s instructions to the teacher and student are commonly scripted to ensure that all students receive the same treatment. In contrast, teachers are trained to differentiate instruction based on their assessment of student needs. The challenge is to incorporate nuance and flexibility into our research studies in the classroom, while at the same time maintaining scientific rigour.

One way to ensure that a research design is sensitive to pressures in a classroom environment is to involve teachers in its conceptualization. Teachers may expose realities of the classroom that researchers are unaware of. Together, teachers and researchers can discuss which parts of a study design can be flexible and which are important to keep consistent across participants. Researchers can also consider an iterative design process. Researchers need to adhere to a finalized protocol for the entirety of data collection. Teachers, however, routinely change how they teach a topic based on their observations and assessments of the students in real time. Researchers may want to consider taking a similar approach. In other words, before finalizing a protocol to use in a study, researchers may consider observing a teacher (and students as they learn) during instruction over an extended period to see how the topic is addressed in a classroom. As researchers gain insight into the teacher and students, they can adapt their protocol to best reflect the realities of the classroom. The final protocol, which must be consistent across learners, will at the very least be sensitive to how the topic is addressed in the classroom.

Social science researchers who study gesture observe naturalistic one-on-one teaching sessions using, for the most part, quantitative methods. However, educators often report that qualitative methods are needed to capture the nuances of the classroom [42,48,49]. Qualitative methods may be needed to develop hypotheses about how gestures can be effectively used in the classroom. But, in the end, we need to be able to apply quantitative methods to our classroom observations to ensure that we are approaching the learning/teaching situation in an unbiased fashion. A mixed methods approach seems best and may be able to triangulate findings and capture the nuances of diverse classrooms and students [50]. Working with teachers to adapt classroom-sensitive teaching methods may require researchers to step out of their comfort zone (and it may also require teachers to develop an appreciation for experimental methods), but it could have a positive, applicable, sustainable influence on education.

Few studies reflect the way in which teachers realistically engage in instruction. One example of researchers who have made an effort to investigate the impact of gesture on instruction in a realistic situation is illustrated in Alibali *et al*. [51]. In this study, an experienced teacher was asked to give a mathethamatics lesson about slopes and intercepts, before and after being shown a tutorial on how to use gesture strategically in mathethamatics instruction. After the tutorial, the teacher increased his rate of gesture and connected more ideas through speech and gesture. Videos of the teacher’s lesson before and after he viewed the tutorial were shown to a group of middle school students, who were later assessed on what they learned. The students who saw the teacher’s instruction after the tutorial (when the instructor increased his rate of gesture production and made more links between his speech and gesture) learned more than the students who saw the video before the tutorial (before the teacher was shown how to capitalize on gesture during instruction). This study provides evidence that, when teachers are shown how to gesture strategically, they can use those gestures to enhance learning outcomes. This study also shows that the impact of strategically gesturing extends to real teachers and real students.

### (b) How can researchers effectively connect with teachers to promote gesture in the classroom?

Efforts have been made in the past to bring researchers and teachers together to improve education. Researchers have conducted projects in schools in an attempt to make their work more directly applicable to classrooms, and they have attempted to disseminate their research outcomes regularly to practitioners [52–54]. At the same time, educators are increasingly interested in finding, discussing and implementing empirical work that focuses on their daily teaching practices [55–59]. Nevertheless, the gap between teachers and researchers remains. To make an impactful connection between teachers and researchers, a long-term, sustainable, bidirectional partnership needs to be established. Because students and teachers are already using gestures in their classrooms [60], gesture-based research may be a viable, low-cost, low-stakes method that can connect teachers and researchers to promote classroom learning.

#### (i) Sustain research–practice partnerships (RPPs)

When researchers conduct studies related to teaching and learning, the work is not often applicable to classroom learning environments [42,43,61]. Even when researchers collect data in classrooms, the data may not provide information about how the instruction can feasibly be implemented in the classroom. Moreover, educators are rarely informed of the outcomes of the experimental experience that took place in their classrooms. One approach to promoting effective, sustained collaboration between researchers and teachers is to establish RPPs [62]. The core elements of RPPs address practice-based questions, encourage shared authority, promote collaborative decision-making among partners, facilitate intentional and structured interactions and endorse original research [62,63]. RPPs establish trust between educators and researchers by promoting collaborative efforts in conceptualizing, designing and executing research aimed at having an actionable outcome [64,65]. RPPs enhance the skills and capabilities of both educators and researchers—educators gain first-hand experience contributing to research, and researchers gain an understanding of the realities of teaching and learning. The ultimate goal of RPPs is to develop applicable, practice-based research initiatives that improve learning outcomes and educational conditions at large [63,66,67].

Based on a survey administered by Nathan *et al*. [60], we know that teachers generally believe that they use spontaneous gestures on a regular basis during instruction and that gestures can be a helpful teaching method. Moreover, as noted earlier, Alibali *et al*. [51] have shown that teachers can learn how to gesture more effectively after watching a simple tutorial (see also Kelly *et al*. [23], who show that teachers can learn to glean information from a student’s gestures more effectively even after a brief hint). These findings establish a basis for initiating partnerships with educators. Researchers can explain to teachers that modifying the gestures they are already producing during instruction could lead to positive outcomes. If teachers then implement this modification in their classrooms, researchers will have the possibility of testing whether changes in teacher gestures have a positive impact on student learning in the classroom. Conversely, researchers can learn from teachers which areas of instruction are particularly problematic in the classroom. Researchers can then suggest ways in which teacher gestures can be modified to improve teaching and learning. Using reports from teachers to guide research questions may promote studies that produce useful outcomes for the classroom. To date, many studies investigating gesture have been focused on mathethamatics, specifically mathethamatics equivalence, but engaging teachers in the conceptualization process via an RPP could help expand the repertoire of gesture-based instruction.

#### (ii) Communities of practice (COP) centred on teacher noticing

Many educators are engaged in communities of practice (COPs) designed to develop the skills needed to improve learning outcomes [68]. COPs are defined as groups of individuals—in this case, teachers—who interact regularly to develop shared resources, experiences and methods of addressing concerns within the field [69]. Teachers engaged in COPs develop skills that often go beyond what is required as part of the curriculum and assessment practices mandated by the district. Some COPs involve the development of reflective practices and *noticing skills* in mathematics [70]. *Teacher noticing* refers to the ability of instructors to attend to the details of their classroom and use their observation to make sense of student thinking [71,72]. For example, Sherin & van Es [73] found that, after mathethamatics teachers in a COP met monthly to reflect on videos of their own instructional practices, they began to notice complex details of the classroom environment that they had not noted in real time. van Es & Sherin [74] suggest that reflective, observational practices not only allow teachers to notice more about the classroom but also help them to make sense of student thinking and develop more relevant and effective instructional responses [75,76]. Similarly, Walkoe *et al*. [77] found that, by training teachers to attend to student non-verbal behaviour, the teachers’ interpretation of their students’ thinking and responses to their students were enriched. After the training, teachers focused less on the correctness of student answers and more on the processes that the students used to come up with their answers. The teachers also responded to students in a more productive way; they asked the students more open-ended follow-up questions that allowed them to gain even more information about their thought processes. Walkoe *et al*. [77] suggest that more support should be implemented within professional development to train teachers to notice non-verbal communication.

Developing a partnership within pre-established COP dedicated to improving mathematics instruction may help researchers to incorporate gestures into the classroom. Once trust is established between researchers and teachers, researchers can make suggestions about how to hone gestures for instruction and propose ways of testing the efficacy of those gestures. Researchers can capitalize on the fact that teachers regularly use gestures during instruction and recognize the importance of gestures in instruction. Creating a partnership with a pre-established COP would be one way to investgiate the effectiveness of transferring gesture-based practices from the lab to the classroom. Together, teachers and researchers could discuss their ideas and routinely adjust protocols to best address their observations.

## 4. Conclusion

All humans gesture, and most of us, including teachers, gesture with little awareness that we are gesturing and that gesture can be a powerful teaching and learning tool. Unintentional gestures have been found to be effective in teaching and learning situations when tested in the lab, but because of the complexities involved in classroom teaching, we need to broaden our methods of inquiry to evaluate gestures’ effectiveness in classroom learning environments. It is clear that gestures can play a powerful role in teaching and learning in one-on-one tutorials. The challenge is to take advantage of this potential to expand gesture’s effectiveness in the classroom.

Before researchers can help teachers reap the benefits of gesture, we must first collaborate with one another to gain an understanding of how gesture-based research can be made more applicable to classrooms. Our goal in this review was twofold: (i) to compile evidence indicating the ways in which gesture can be beneficial as a tool during mathematics instruction, and (ii) to suggest areas where researchers can modify their traditional methods to promote gesture-based studies that are more applicable to the daily lives of teachers and students. By recognizing the differences in approach that researchers and teachers take to address questions, and by capitalizing on the unique strengths of each group, we can create partnerships and COP that allow us to collectively work towards using gestures to promote learning. Gesture research offers a low-cost, low-stakes way of bringing researchers and teachers together to improve learning.

## Data Availability

This article has no additional data.

## References

[1] Goldin-Meadow S . 2003 Hearing gesture: how our hands help us think. Cambridge, MA: Belknap Press of Harvard University Press.

[2] Goldin-Meadow S . 2023 Thinking with your hands: the surprising science behind how gestures shape our thoughts, pp. 49–50. New York, NY: Basic Books.

[3] Kendon A . 2004 Gesture visible action as utterance. In Gesture: visible action as utterance. Cambridge, UK: Cambridge University Press. (10.1017/CBO9780511807572). See https://www.cambridge.org/core/product/identifier/9780511807572/type/book.

[4] McNeill D . 1992 Hand and mind. Chicago, IL: University of Chicago Press.

[5] Wakefield E , Novack MA , Congdon EL , Franconeri S , Goldin-Meadow S . 2018 Gesture helps learners learn, but not merely by guiding their visual attention. Dev. Sci. **21** , e12664. (10.1111/desc.12664) 29663574 PMC6191377

[6] Cook SW , Yip TKY , Goldin-Meadow S . 2010 Gesturing makes memories that last. J. Mem. Lang. **63** , 465–475. (10.1016/j.jml.2010.07.002) 21731176 PMC3124384

[7] Goldin-Meadow S , Nusbaum H , Kelly SD , Wagner S . 2001 Explaining math: gesturing lightens the load. Psychol. Sci. **12** , 516–522. (10.1111/1467-9280.00395) 11760141

[8] Ping R , Goldin-Meadow S . 2010 Gesturing saves cognitive resources when talking about non-present objects. Cogn. Sci. **34** , 602–619. (10.1111/j.1551-6709.2010.01102.x) 21564226 PMC3733275

[9] Beaudoin-Ryan L , Goldin-Meadow S . 2014 Teaching moral reasoning through gesture. Dev. Sci. **17** , 984–990. (10.1111/desc.12180) 24754707 PMC4207722

[10] Mayer RE . 2009 Multimedia learning, 2^nd^ edition. New York, NY: Cambridge University Press. (10.1017/CBO9780511811678)

[11] Sullivan JV . 2018 Learning and embodied cognition: a review and proposal. Psychol. Learn. Teach. **17** , 128–143. (10.1177/1475725717752550)

[12] Church RB , Goldin-Meadow S . 1986 The mismatch between gesture and speech as an index of transitional knowledge. Cognition **23** , 43–71. (10.1016/0010-0277(86)90053-3) 3742990

[13] Piaget J . 1965 The child’s conception of number. New York, NY: W.W. Norton and Company.

[14] Adelman C . 2006 The Toolbox Revisited: paths to degree completion from high school through college. Washington, DC: US Department of Education.

[15] Atanda R . 1999 Do gatekeeper courses expand education options? Washington, DC: National Center for Education Statistics.

[16] Hansen M . 2014 Characteristics of Schools Successful in STEM: Evidence from Two States’ Longitudinal Data. J. Educ. Res. **107** , 374–391. (10.1080/00220671.2013.823364)

[17] Riley RW . 1997 Mathematics equals opportunity. Washington, DC: US Department of Education.

[18] Perry M , Breckinridge Church R , Goldin-Meadow S . 1988 Transitional knowledge in the acquisition of concepts. Cogn. Dev. **3** , 359–400. (10.1016/0885-2014(88)90021-4)

[19] Alibali MW , Goldin-Meadow S . 1993 Modeling learning using evidence from speech and gesture. In Proceedings of the Fifteenth Annual Conference of the Cognitive Science Society, pp. 203–208. Hillsdale, NJ: Erlbaum.

[20] Pine KJ , Lufkin N , Messer D . 2004 More gestures than answers: children learning about balance. Dev. Psychol. **40** , 1059–1067. (10.1037/0012-1649.40.6.1059) 15535756

[21] Alibali MW , Flevares LM , Goldin-Meadow S . 1997 Assessing knowledge conveyed in gesture: do teachers have the upper hand? J. Educ. Psychol. **89** , 183–193. (10.1037//0022-0663.89.1.183)

[22] Goldin‐Meadow S , Sandhofer CM . 1999 Gesture conveys substantive information about a child’s thoughts to ordinary listeners. Dev. Sci. **2** , 67–74. (10.1111/1467-7687.00056)

[23] Kelly SD , Singer M , Hicks J , Goldin-Meadow S . 2002 A helping hand in assessing children’s knowledge: instructing adults to attend to gesture. Cogn. Instr. **20** , 1–26. (10.1207/S1532690XCI2001_1)

[24] Cook SW , Goldin-Meadow S . 2006 The role of gesture in learning: do children use their hands to change their minds? J. Cogn. Dev. **7** , 211–232. (10.1207/s15327647jcd0702_4)

[25] Novack MA , Congdon EL , Hemani-Lopez N , Goldin-Meadow S . 2014 From action to abstraction: using the hands to learn math. Psychol. Sci. **25** , 903–910. (10.1177/0956797613518351) 24503873 PMC3984351

[26] Cook SW , Mitchell Z , Goldin-Meadow S . 2008 Gesturing makes learning last. Cognition **106** , 1047–1058. (10.1016/j.cognition.2007.04.010) 17560971 PMC2265003

[27] Carrazza C , Wakefield EM , Hemani-Lopez N , Plath K , Goldin-Meadow S . 2021 Children integrate speech and gesture across a wider temporal window than speech and action when learning a math concept. Cognition **210** , 104604. (10.1016/j.cognition.2021.104604) 33548851

[28] Singer MA , Goldin-Meadow S . 2005 Children learn when their teachers’ gestures and speech differ. Psychol. Sci. **16** , 85–89. (10.1111/j.0956-7976.2005.00786.x) 15686572

[29] Congdon EL , Novack MA , Brooks N , Hemani-Lopez N , O’Keefe L , Goldin-Meadow S . 2017 Better together: simultaneous presentation of speech and gesture in math instruction supports generalization and retention. Learn. Instr. **50** , 65–74. (10.1016/j.learninstruc.2017.03.005) 29051690 PMC5642925

[30] Goldin-Meadow S , Singer MA . 2003 From children’s hands to adults’ ears: gesture’s role in teaching and learning. Dev. Psychol. **39** , 509–520. (doi: 10.1037/0012-1649.39.3.509). 12760519 10.1037/0012-1649.39.3.509

[31] Krönke KM , Mueller K , Friederici AD , Obrig H . 2013 Learning by doing? The effect of gestures on implicit retrieval of newly acquired words. Cortex. **49** , 2553–2568. (10.1016/j.cortex.2012.11.016) 23357203

[32] Ping RM , Goldin-Meadow S , Beilock SL . 2014 Understanding gesture: is the listener’s motor system involved J. Exp. Psychol. Gen. **143** , 195–204. (10.1037/a0032246) 23565671 PMC3759547

[33] Dargue N , Sweller N , Jones MP . 2019 When our hands help us understand: a meta-analysis into the effects of gesture on comprehension. Psychol. Bull. **145** , 765–784. (10.1037/bul0000202) 31219263

[34] Goldin‐Meadow S , Levine SC , Zinchenko E , Yip TK , Hemani N , Factor L . 2012 Doing gesture promotes learning a mental transformation task better than seeing gesture. Dev. Sci. **15** , 876–884. (10.1111/j.1467-7687.2012.01185.x) 23106741 PMC3490223

[35] Congdon EL , Novack MA , Wakefield EM , Hemani-Lopez N , Goldin-Meadow S . 2024 Learners’ spontaneous gestures before a math lesson predict the efficacy of seeing versus doing gesture during the lesson. In press. Cogn. Sci. **48** , e13479. (10.1111/cogs.13479) 38980965 PMC13014393

[36] Wakefield EM , Hall C , James KH , Goldin-Meadow S . 2018 Gesture for generalization: gesture facilitates flexible learning of words for actions on objects. Dev. Sci. **21** , e12656. (10.1111/desc.12656) 29542238

[37] Chu M , Meyer A , Foulkes L , Kita S . 2014 Individual differences in frequency and saliency of speech-accompanying gestures: the role of cognitive abilities and empathy. J. Exp. Psychol. Gen. **143** , 694–709. (10.1037/a0033861) 23915128 PMC3970852

[38] Gillespie M , James AN , Federmeier KD , Watson DG . 2014 Verbal working memory predicts co-speech gesture: evidence from individual differences. Cognition **132** , 174–180. (10.1016/j.cognition.2014.03.012) 24813571 PMC4066192

[39] Pouw WTJL , Mavilidi MF , van Gog T , Paas F . 2016 Gesturing during mental problem solving reduces eye movements, especially for individuals with lower visual working memory capacity. Cogn. Process. **17** , 269–277. (10.1007/s10339-016-0757-6) 26993293

[40] Aldugom M , Fenn K , Cook SW . 2020 Gesture during math instruction specifically benefits learners with high visuospatial working memory capacity. Cogn. Res. Princ. Implic. **5** , 27. (10.1186/s41235-020-00215-8) 32519045 PMC7283399

[41] Broaders SC , Cook SW , Mitchell Z , Goldin-Meadow S . 2007 Making children gesture brings out implicit knowledge and leads to learning. J. Exp. Psychol. Gen. **136** , 539–550. (10.1037/0096-3445.136.4.539) 17999569

[42] Gore JM , Gitlin AD . 2004 [RE]Visioning the academic–teacher divide: power and knowledge in the educational community. Teach. Teach. **10** , 35–58. (10.1080/13540600320000170918)

[43] Broekkamp H , van Hout-Wolters B . 2007 The gap between educational research and practice: a literature review, symposium, and questionnaire. Educ. Res. Eval. **13** , 203–220. (10.1080/13803610701626127)

[44] McIntyre D . 2005 Bridging the gap between research and practice. Cambridge J. Educ. **35** , 357–382. (10.1080/03057640500319065)

[45] Bates R . 2002 The impact of educational research: alternative methodologies and conclusions. Res. Pap. Educ. **17** , 403–408. (10.1080/0267152022000031379)

[46] Berliner DC . 2002 Comment: Educational research: The Hardest Science of All. Educ. Res. **31** , 18–20. (10.3102/0013189X031008018)

[47] Burkhardt H , Schoenfeld AH . 2003 Improving educational research: toward a more useful, more influential, and better-funded enterprise. Educ. Res. **32** , 3–14. (10.3102/0013189X032009003)

[48] Seidman I . 2006 Interviewing as qualitative research: a guide for researchers in education and the social sciences. New York, NY: Teachers College Press.

[49] Shkedi A . 1998 Teachers’ attitudes towards research: a challenge for qualitative researchers. Int. J. Qual. Stud. Educ. **11** , 559–577. (10.1080/095183998236467)

[50] Creswell JW , Plano Clark VL . 2007 Designing and conducting mixed methods research. London, UK: Sage Publications.

[51] Alibali MW , Nathan MJ , Church RB , Wolfgram MS , Kim S , Knuth EJ . 2013 Teachers’ gestures and speech in mathematics lessons: forging common ground by resolving trouble spots. ZDM **45** , 425–440. (10.1007/s11858-012-0476-0)

[52] Biddle BJ , Saha LJ . 2006 How principals use research. Educ. Leadersh. **63** , 72–77.

[53] Malin JR , Paralkar VK . 2017 Knowledge mobilization in education: the Marshall Memo case. In Annual Meeting of the American Educational Research Association, San Antonio, TX.

[54] Behrstock-Sherratt E , Drill K , Miller S . 2011 Is the supply in demand? Exploring how, when, and why teachers use research. Washington, DC: American Institutes for Research.

[55] Corcoran TB , McVay S , Riordan K . 2003 Getting it right: the MISE approach to professional development. Consortium for Policy Research in Education, University of Pennsylvania Graduate School of Education. See http://repository.upenn.edu/cpre_ researchreports/42.

[56] Farley-Ripple EN . 2012 Research use in school district central office decision making: a case study. Educ. Manag. Adm. Leadersh. **40** , 786–806. (10.1177/1741143212456912)

[57] Farley-Ripple E , May H , Karpyn A , Tilley K , McDonough K . 2018 Rethinking connections between research and practice in education: a conceptual framework. Educ. Res. **47** , 235–245. (10.3102/0013189X18761042)

[58] Newman J , Cherney A , Head BW . 2016 Do policy makers use academic research? Reexamining the “Two Communities” theory of research utilization. Public Adm. Rev. **76** , 24–32. (10.1111/puar.12464)

[59] Massell D , Goertz ME , Barnes CA . 2012 State education agencies’ acquisition and use of research knowledge for school improvement. Peabody J. Educ. **87** , 609–626. (10.1080/0161956X.2012.723506)

[60] Nathan MJ , Yeo A , Boncoddo R , Hostetter AB , Alibali MW . 2019 Teachers’ attitudes about gesture for learning and instruction. Gesture **18** , 31–56. (10.1075/gest.00032.nat)

[61] Vanderlinde R , van Braak J . 2010 The gap between educational research and practice: views of teachers, school leaders, intermediaries and researchers. Br. Educ. Res. J. **36** , 299–316. (10.1080/01411920902919257)

[62] Coburn CE , Penuel WR , Geil KE . 2013 Practice partnerships: a strategy for Leveraging research for educational improvement in school districts. New York, NY: William T. Grant Foundation.

[63] Coburn CE , Penuel WR . 2016 Research–practice partnerships in education. Educ. Res. **45** , 48–54. (10.3102/0013189X16631750)

[64] Goldstein H , McKenna M , Barker RM , Brown TH . 2019 Research–practice partnership: application to implementation of multitiered system of supports in early childhood education. Perspect. ASHA Spec. Interest Groups **4** , 38–50. (10.1044/2018_PERS-ST-2018-0005)

[65] Henrick EC , Cobb P , Penuel WR , Jackson K , Clark T . 2017 Assessing research-practice partnerships: five dimensions of effectiveness. New York, NY: William T. Grant Foundation.

[66] Donovan MS . 2013 Generating improvement through research and development in education systems. Science **340** , 317–319. (10.1126/science.1236180) 23599484

[67] Tseng V . 2012 Partnerships: shifting the dynamics between research and practice. vol. 76. New York, NY: William T. Grant Foundation.

[68] Wineburg SS , Grossman P . 1998 Creating a community of learners among high school teachers. Phi Delta Kappan **79** , 350. (10.1177/003172171407900707)

[69] Lave J , Wenger E . 1991 Situated learning: legitimate peripheral participation. Cambridge, UK: Cambridge University Press. (10.1017/CBO9780511815355)

[70] Sherin M , Jacobs V , Philipp R . 2010 Mathematics teacher noticing: seeing through teachers’ eyes. Abingdon, UK: Routledge. (10.4324/9780203832714)

[71] Van Es EA , Sherin MG . 2002 Learning to notice: scaffolding new teachers’ interpretations of classroom interactions. J. Technol. Teach. Educ. **10** , 571–596. (10.1080/15391523.2002.10782312)

[72] van Es EA , Sherin MG . 2008 Mathematics teachers’ “learning to notice” in the context of a video club. Teach. Teach. Educ. **24** , 244–276. (10.1016/j.tate.2006.11.005)

[73] Sherin M , van Es E . 2005 Using video to support teachers’ ability to notice classroom interactions. J. Technol. Teach. Educ. **13** , 475–491. (10.1080/10508400500367682)

[74] van Es EA , Sherin MG . 2021 Expanding on prior conceptualizations of teacher noticing. ZDM Math. Educ. **53** , 17–27. (10.1007/s11858-020-01211-4)

[75] Santagata R , Yeh C . 2014 Learning to teach mathematics and to analyze teaching effectiveness: evidence from a video- and practice-based approach. J. Math. Teacher Educ. **17** , 491–514. (10.1007/s10857-013-9263-2)

[76] Ulusoy F . 2020 Prospective teachers’ skills of attending, interpreting and responding to content-specific characteristics of mathematics instruction in classroom videos. Teach. and Teach. Educ. **94** , 103103. (10.1016/j.tate.2020.103103)

[77] Walkoe J , Williams-Pierce CC , Flood VJ , Walton M . 2023 Toward professional development for multimodal teacher noticing. J. Res. Math. Educ. **54** , 279–285. (10.5951/jresematheduc-2020-0326)

